# Quality of Life, Fatigue, and Physical Symptoms Post-COVID-19 Condition: A Cross-Sectional Comparative Study

**DOI:** 10.3390/healthcare11111660

**Published:** 2023-06-05

**Authors:** Maha M. AlRasheed, Sinaa Al-Aqeel, Ghada I. Aboheimed, Noura M. AlRasheed, Norah Othman Abanmy, Ghadeer Abdulaziz Alhamid, Hadeel Mohammed Alnemari, Saad Alkhowaiter, Abdullah Rashed Alharbi, Fowad Khurshid, Khaled Trabelsi, Haitham A. Jahrami, Ahmed S. BaHammam

**Affiliations:** 1 Department of Clinical Pharmacy, College of Pharmacy, King Saud University, Riyadh 11451, Saudi Arabia; salageel@ksu.edu.sa (S.A.-A.); ghaboheimed@ksu.edu.sa (G.I.A.); nabanmy@ksu.edu.sa (N.O.A.); 438201870@student.ksu.edu.sa (G.A.A.); 439200475@student.ksu.edu.sa (H.M.A.); fowad.khurshid@mangalayatan.edu.in (F.K.); 2 General Administration of School Health, Ministry of Health, Riyadh 11451, Saudi Arabia; nomalrasheed@moh.gov.sa; 3 Department of Medicine, Gastroenterology Division, College of Medicine, King Saud University, Riyadh 11451, Saudi Arabia; salkhowaiter@ksu.edu.sa; 4 Department of Medicine, Pulmonary Division, College of Medicine, King Saud University, Riyadh 11451, Saudi Arabia; rabdullah@ksu.edu.sa; 5 High Institute of Sport and Physical Education of Sfax, University of Sfax, Sfax 3000, Tunisia; khaled.trabelsi@isseps.usf.tn; 6 Research Laboratory: Education, Motricity, Sport and Health, EM2S, LR19JS01, University of Sfax, Sfax 3000, Tunisia; 7 Government Hospitals, Manama 323, Bahrain; hjahrami@health.gov.bh; 8 College of Medicine and Medical Sciences, Arabian Gulf University, Manama 323, Bahrain; 9 Department of Medicine, College of Medicine, University Sleep Disorders Center, King Saud University, Riyadh 11451, Saudi Arabia; ashammam@ksu.edu.sa

**Keywords:** post-acute COVID-19 syndrome, quality of life, fatigue severity scale, SF-36, fatigue

## Abstract

The magnitude of post-COVID-19 syndrome was not thoroughly investigated. This study evaluated the quality of life and persistence of fatigue and physical symptoms of individuals post-COVID-19 compared with noninfected controls. The study included 965 participants; 400 had previous COVID-19 disease and 565 controls without COVID-19. The questionnaire collected data on comorbidities, COVID-19 vaccination, general health questions, and physical symptoms, in addition to validated measures of quality of life (SF-36 scale), fatigue (fatigue severity scale, FSS), and dyspnea grade. COVID-19 participants complained more frequently of weakness, muscle pain, respiratory symptoms, voice disorders, imbalance, taste and smell loss, and menstrual problems compared to the controls. Joint symptoms, tingling, numbness, hypo/hypertension, sexual dysfunction, headache, bowel, urinary, cardiac, and visual symptoms did not differ between groups. Dyspnea grade II–IV did not differ significantly between groups (*p* = 0.116). COVID-19 patients scored lower on the SF-36 domains of role physical (*p* = 0.045), vitality (*p* < 0.001), reported health changes (*p* < 0.001), and mental-components summary (*p* = 0.014). FSS scores were significantly higher in COVID-19 participants (3 (1.8–4.3) vs. 2.6 (1.4–4); *p* < 0.001). COVID-19 effects could persist beyond the acute infection phase. These effects include changes in quality of life, fatigue, and persistence of physical symptoms.

## 1. Introduction

Coronavirus 2 (SARS-CoV-2) infected millions worldwide, with thousands of deaths in the last few years. Many COVID-19 patients experience new, recurring, or ongoing symptoms that last beyond the period of active infection. These symptoms are referred to as a long-COVID or post-COVID-19 condition. This condition is defined as new or persistent symptoms occurring in patients with prior confirmed or possible COVID-19 and cannot be explained by an alternate diagnosis [1,2]. The pooled global prevalence of post-COVID-19 was estimated to be 43%, with a higher prevalence in hospitalized patients of 54% [3]. The pooled prevalence of post-COVID-19 in children and adolescents was estimated to be 25.24% [4].

The post-COVID-19 condition impacts many organ systems, including pulmonary, hematologic, cardiovascular, neuropsychiatric, renal, endocrine, and gastrointestinal [5,6]. The common symptoms identified in post-COVID-19 patients include fatigue, cough, dyspnea, chest pain/tightens, headache, sleep disturbance, and mental health problems [4,7,8,9]. Fatigue was the most described post-COVID-19 symptom. Few meta-analyses estimated the prevalence of post-COVID fatigue with varying prevalence rates based on inclusion and exclusion criteria for each study. The prevalence of fatigue 3 to 6 months after COVID-19 symptom onset or hospital discharge was 38% [9] and 28% after one year [10]. A higher prevalence of 64% was reported in another meta-analysis [7]. One systematic review found a prevalence of fatigue, dyspnea, sleep disorder, and myalgia among individuals with confirmed acute COVID-19 and were 41%, 31%, 30%, and 22%, respectively, at >12-month follow-up [11]. Another review found the most prevalent post-COVID-19 symptoms were fatigue and dyspnea with a pooled prevalence of 42% (27–58%) followed by sleep disturbance 28% (14–45%), cough 25% (10–44%), fever 21% (4–47%), myalgia 17% (2–41%), chest pain 11% (5–20%), and headache 9% (2–20%) [12]. The causes of post-COVID-19 symptoms are unknown, and several pathophysiological mechanisms, including immune system abnormalities and antigen production or perseverance, could be implicated [6,13].

The impact of COVID-19 extends beyond clinical symptoms to health-related quality of life (HRQoL) and economic and societal impacts, as evidence suggests [6,14]. Several systematic reviews investigated the acute and long-term impact of COVID-19 on HRQoL. One systematic review on COVID-19 estimated the pooled prevalence of decreased HRQol in an average of 52% of post-COVID-19 patients [9]. A meta-analysis estimated the pooled prevalence of poor quality of life among post-COVID-19 patients using the EQ-VAS questionnaire was 59%, while the pooled prevalence using the EQ-5Q-5L questionnaire showed that 42% of post-COVID-19 patients had pain/discomfort, 38% had anxiety/depression, 36% had problems with mobility, 28% had problems with usual activities, and 8% had personal-care problems [7].

The majority of previous studies examined COVID-19’s impact on quality of life (HRQoL) through cross-sectional data, primarily from China, Europe, or the United States. Moreover, the focus was mainly on HRQoL among hospitalized or previously hospitalized COVID-19 patients, neglecting a thorough investigation of HRQoL in nonhospitalized patients [15]. To fill this gap, it is essential to evaluate the impact of COVID-19 in other countries. Additionally, comparing the HRQoL of individuals infected with COVID-19 (hospitalized and nonhospitalized) to that of unaffected controls would provide new insights into understanding the virus’s impact. Therefore, the objective of this study was to assess the impact of COVID-19 on quality of life, as well as investigate the persistence of fatigue and physical symptoms. The study aimed to compare the quality of life and persistence of fatigue and physical symptoms of individuals with COVID-19 compared with controls (with no COVID-19) and evaluated their risk factors. We hypothesized that individuals who have recovered from COVID-19 report lower quality of life, higher levels of fatigue, and a higher frequency of physical symptoms compared to noninfected controls.

## 2. Patients and Methods

### 2.1. Design

This cross-sectional study compared individuals with previous COVID-19 to individuals with no prior COVID-19. The long-term effects of COVID-19 were compared between the two groups regarding the quality of life, fatigue, and physical symptoms. The participants self-reported the time elapsed from infection to the reported measures at the following intervals (<6 months, 6–12 months, 12–18 months, 18–24 months, and >24 months).

### 2.2. Sample

Participants aged 18 years and older capable of reading and understanding the questionnaire were eligible to participate. A convenience sampling technique was employed, and invitations to participate in the study were delivered through Twitter and WhatsApp. Individuals from different Arab countries were invited to participate. The questionnaire was available in Arabic and English for participants to select their preferred language. The online survey was available from 4 October to 26 October 2022. No monetary or nonmonetary rewards were provided for participation.

### 2.3. Sample Size Calculation

The sample size was calculated to be 384 with the expected outcome (poor quality of life) in 50% of participants with previous COVID-19, the confidence level was set to 95%, and the margin of error was 5%. If the response rate is 33%, we need to send the survey to 1152 participants to reach the target sample size.

### 2.4. The Questionnaire

The questionnaire was tested on experts and volunteers, and after minor adjustments, the online questionnaire was constructed using Google Forms. The first page of the survey contained a detailed description of the study, the ethical approval number, the lead author’s name and email for any questions, the expected time to complete the questionnaire, the voluntary nature of participation, and the freedom to quit the questionnaire at any time. The participant must consent to participate to proceed to the questionnaire. Answering all questions was mandatory to avoid missing responses. We provided opt-out options such as “I don’t know” as needed.

The questionnaire collected information about the following: demographics, presence of comorbidities, COVID-19 vaccination status, history of a positive test for COVID-19, general health questions, and physical symptoms at the time of answering the questionnaire, in addition to validated measures of quality-of-life and fatigue and dyspnea grade using the Modified Medical Research Council Dyspnea scale [16].

To understand the impact of the number of SARS-CoV-2 infections and the time since the last infection, those who answered “Yes” to the question “Have you tested positive for COVID-19 previously?” answered additional questions related to the number of times they tested positive, the last time to test positive for the infection (<6 months, 6–12 months, 12–24 months or >24 months), as well as the onset of infection in relation to the vaccination, and COVID-19 symptoms. 

The questionnaire was available from 4 October to 26 October 2022 through the following link: https://docs.google.com/forms/d/e/1FAIpQLSc8MrU8AjnM9HqKZUTc0LURb0yfo3QU7bZ3Qo2hmRdFE6VeeA/viewform (accessed on 4 October 2022).

### 2.5. Measures

#### 2.5.1. The 36-Item Short Form Survey (SF-36)

SF-36 is a generic and easily administered quality-of-life measure composed of 36 items under eight domains [17]: Physical functioning (10 items), role limitations due to physical health (4 items), role limitations due to emotional problems (3 items), energy fatigue (4 items), emotional well-being (5 items), social functioning (2 items), pain (2 items), and general health (5 items). The SF-36 scores were calculated according to RAND Corporation scoring instructions. First, the original response categories were recoded on a 0–100 range, with a lower score defining a less favorable health state. Then, the average scores for each of the eight scales were calculated. (https://www.rand.org/health-care/surveys_tools/mos/36-item-short-form/scoring.html (accessed on 4 May 2023)).

Two more scores were calculated; physical (PCS) and mental (MCS) components summary. Score calculations were performed using Stata 17 statistical software (Stata Corp, College Station, TX, USA). The Arabic version was obtained from the RAND Corporation website. The reliability of the Arabic version among the Saudi population was previously demonstrated [18].

#### 2.5.2. Fatigue Severity Scale

The fatigue severity scale (FSS) is a self-reported measure of how fatigue interferes with certain activities [19]. It consists of 9 statements, and participants indicate the extent to which they agree or disagree by selecting a number from 1 to 7, where 1 indicates strong disagreement and 7 indicates strong agreement. The FSS is scored on a 7-point scale, with 9 being the minimum and 63 being the maximum score [19]. The higher the score, the greater the fatigue severity. The Arabic version of FSS showed acceptable reliability (intraclass correlation coefficient model 2,1 = 0.80) and internal consistency (Cronbach’s alpha = 0.84) in the Saudi population [20].

#### 2.5.3. Data Analysis

We stratified the participants into two groups. Group 1 included participants with self-reported positive COVID-19 tests, and Group 2 was without positive COVID-19 tests.

A descriptive analysis was used to present our results. Normality was assessed with the Shapiro–Wilk test and histograms. Continuous data were expressed as median (25th and 75th percentiles) and compared between COVID-19 and non-COVID-19 participants with the Wilcoxon test or the *t*-test. Comparison of continuous data among three or more subgroups (i.e., duration from acute COVID-19 and the SF-36 domains) was performed using the one-way Analysis of Variance (ANOVA) test if the equal variance was achieved or the Kruskal–Wallis (nonparametric ANOVA) in the case of nonequal variance. Post hoc analysis was done using the Bonferroni test after ANOVA and Dunn’s test after the Kruskal–Wallis test when required. Nominal data were expressed as frequencies and percentages and compared with the Chi-squared or Fisher exact test. Factors affecting the physical and mental-component summaries of the SF-36 questionnaire were tested with linear regression, and the assumptions of linear regression were tested with the normal distribution of the residuals (with histograms) and residual versus fitted plots. Collinearity was assessed with the variance inflation factor (VIF), and all included variables had VIF. The correlation between the FSS and the SF-36 score was evaluated with Spearman correlation, and factors affecting FSS were evaluated with quantile regression. Data were analyzed using Stata 17 (Stata Corp, College Station, TX, USA) and a *p*-value of less than 0.05 was considered statistically significant. 

#### 2.5.4. Ethics

The study was conducted following the declaration of Helsinki’s ethical principles and approved by King Saud Medical City Institutional Review Board (E-22-6729). Electronic informed consent was obtained from the respondents before starting to answer the questionnaire. In this research, participation was entirely voluntary, and participants were free to leave at any time.

## 3. Results

### 3.1. Study Participants

The study included 965 participants; 400 had previous COVID-19 and 565 controls. There was no difference in gender distribution between COVID-19 and non-COVID-19 participants. (Table 1) Several baseline characteristics were significantly different between the groups. The most common age category for COVID-19 participants was between 36 and 45 years versus 26–35 years for controls. BMI was significantly higher in the COVID-19 group, and most participants in the COVID-19 group were married and employed. The number of unvaccinated individuals was significantly higher in the control group. There was no difference in comorbidities between groups (Table 1).

### 3.2. Description of COVID-19 Participants

Among the COVID-19 participants, 328 (82%) had the SARS-CoV-2 infection once, and 72 (18%) more than once. The last infection was less than six months in 85 participants (21.25%), 6–12 months in 151 (37.75%), 12–18 months in 60 (15%), 18–24 months in 46 (11.50%), and more than 24 months in 58 (14.50%). Infection occurred before the first dose/while unvaccinated in 139 (34.75%), after the first dose in 63 (15.75%), after the second dose in 119 (29.75%), and after the booster dose in 79 (19.75%). The most common presenting acute symptoms were fever (*n* = 249, 62.25%) followed by cough and loss of taste or smell (*n* = 186, 46.5%, for both), and 134 participants had shortness of breath (33.5%). Twenty-one participants (5.25%) required hospitalization.

### 3.3. Current Physical Symptoms

The current physical symptoms respondents experienced when answering the questionnaire were compared between COVID-19 participants and controls. COVID-19 participants complained more frequently of weakness, muscle pain, respiratory symptoms, voice disorders, imbalance, taste and smell loss, and menstrual problems. Joint symptoms, tingling, numbness, hypo/hypertension, sexual dysfunction, headache, bowel, urinary, cardiac, and visual symptoms did not differ between groups. Dyspnea grade II-IV did not differ significantly between groups (Table 2).

Weakness was highest among those who had COVID-19 less than six months ago and lowest in those who had COVID-19 disease more than 24 months ago (*p* = 0.028). There were no significant differences in other symptoms concerning times from COVID-19.

### 3.4. Quality of Life

Quality of life using the SF-36 questionnaire was compared between COVID-19 and controls. The difference was statistically significant for role physical domain, role emotional domain, vitality domain, reported health change, and mental components (Table 3).

Participants who were infected more than once had lower mean SF-36 scores compared to those with one-time infections. The difference was statistically significant in the following domains: role physical, general health, role emotional, and mental-component summary (Table A1). The SF-36 scores were compared in relation to the time since the last infection in COVID-19 participants (<6 months, 6–12 months, 12–24 months, or >24 months), and there was no difference in all domains among different times from infection. (Table A2).

Factors affecting the physical-component and mental-component summaries were evaluated in all participants. The physical-component summary was significantly higher in males, while it was inversely related to body mass index and the higher age category (Table A3). The mental-component summary was significantly higher in males and high age categories, while COVID-19 infection, smoking, highest education degree, and diabetes mellitus negatively affected the mental-component summary (Table A3).

Among post-COVID-19 participants, weakness, vision problems, urinary symptoms, headache, and joint stiffness negatively affected the physical-component summary, while weakness and headache negatively affected the mental-component summary (Table A4).

COVID-19 participants presenting with shortness of breath were significantly associated with lower mental and physical components summaries (Table A5).

### 3.5. Fatigue Severity Scale

**Figure 1:**
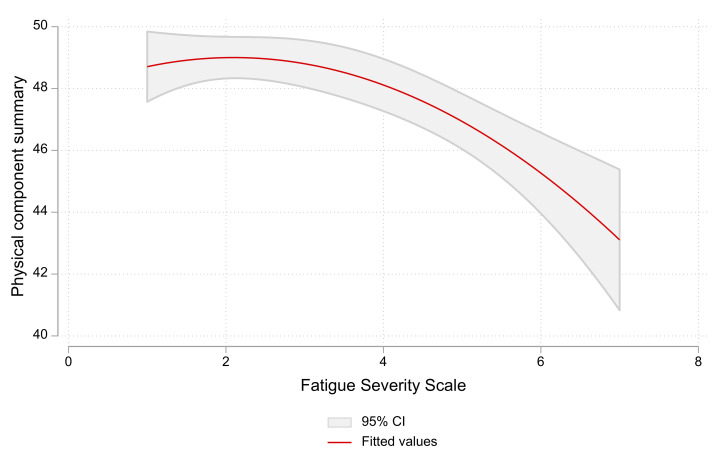
The relation between fatigue severity scale and physical-component summary of SF-36 scores in all participants. A higher fatigue severity scale is associated with a lower physical-component summary of SF-36.

The FSS scores were significantly higher in COVID-19 participants (3 (1.8–4.3) vs. 2.6 (1.4–4); *p* < 0.001). There was no significant difference in FSS according to the time from infection; it was 3.2 (1.8–4.2), 3 (1.9–4.3), 3 (2.1–4.3), 2.9 (1.9–4.6) and 2.9 (1.7–4) for infection occurring <6 months, 6–12 months, 12–18 months, 18–24 months, and >24 months, respectively. There was a significant negative correlation between the FSS score and physical-component summary of the SF-36 score in all participants (r: −0.109, *p* = 0.001), and this relation was maintained in non-COVID-19 participants (r = −0.124, *p* = 0.003), not in COVID-19 participants (r = −0.08, *p* = 0.085) (Figure 1).

**Figure 2:**
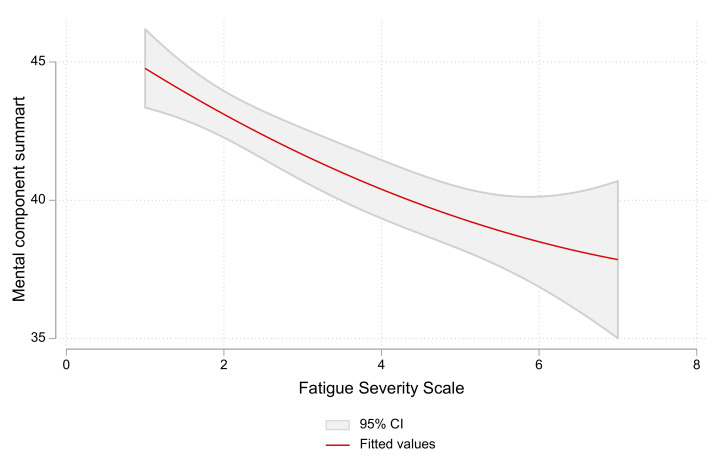
The relation between the fatigue severity scale and mental-component summary of the SF-36 scores in all participants. Higher fatigue severity scale is associated with a lower mental-component summary of SF-36.

There was a negative correlation between the FSS score and the mental-component summary of the SF-36 score in both groups (r = −0.186, *p* ˂ 0.001), and this relation was maintained in non-COVID-19 participants (r = −0.212, *p* ˂ 0.001) and COVID-19 participants (r = −0.132, *p* = 0.008) (Figure 2).

The FSS score was significantly higher in participants with cardiovascular disease, COVID-19, and those living outside Saudi Arabia (Table A6).

## 4. Discussion

In this study, we assessed the effect of COVID-19 on quality of life and the persistence of fatigue and physical symptoms. We analyzed responses to an online survey of individuals with previous COVID-19 disease (*n* = 400) and controls with no COVID-19 disease (*n* = 565). Although the SF-36 scores were slightly higher in non-COVID-19 participants for many domains, the difference was statistically significant only for role physical, role emotional, and vitality domains that reported health change and mental components. Respondents infected more than once had statistically significantly lower mean SF-36 scores than those with one-time infection in the following domains: role physical, role emotional, general health, and the mental-components summary. FSS scores were higher in respondents with COVID-19, indicating a greater fatigue severity than in the control group. The FSS scores were negatively correlated with the physical and mental components of the SF-36. Our study evaluated several aspects of post-COVID-19 (quality of life, fatigue, and physical symptoms) in COVID-19 participants from Saudi Arabia and other Arab countries and compared these domains to controls.

Our findings indicated that COVID-19 negatively impacted certain SF-36 domains more than others, and this is comparable to findings from previous research. Angarita-Fonseca and associates reported that the most common post-COVID symptoms in Latin America were fatigue, sleep problems, headaches, muscle or joint pain, and dyspnea with exertion [21]. Chen and colleagues compared the quality of life of COVID-19 patients in China one month after hospital discharge to that of normal controls. They found that patients have lower physical function, social function, and role physical scores and higher body pain, general health, and vitality scores [3]. Three months after recovery from acute COVID-19 infection, nonhospitalized patients with mild COVID-19 disease reported significantly worse health status on most subscales of the SF-36 compared with patients after inpatient treatment for critical, severe, and moderate COVID-19 disease, and the differences were statistically significant for physical and social functioning, energy/fatigue, and pain [10]. A longitudinal UK-based study comparing HRQoL of nonhospitalized COVID-19 with controls at baseline and six months using an EQ-5D questionnaire reported that cases have more problems with mobility and doing usual activities than controls. In comparison, controls have more problems with pain/discomfort and anxiety/depression domains than cases, but the differences were not significant [22].

On the other hand, a study reported worsened HRQoL in all SF-36 dimensions for moderate and severe COVID-19 patients two to three months after hospital discharge compared to the controls [23] and reported worsening HRQoL in all SF-36 dimensions. The differences in COVID-19 impact on HRQoL could be attributed to heterogeneity in the study design, such as study timing (during the early months of the pandemic with extreme uncertainty versus the postvaccination stage), the severity of COVID-19 infection, time of HRQoL measurement, vaccination status, and sociodemographic characteristics of participants. However, the percentage of respondents with three doses of vaccination is high in both groups because, in February 2022, the booster dose became mandatory in Saudi Arabia for adults who received the second dose of the vaccine eight months ago. 

Our findings showed that factors associated with lower SF-36 scores were older age, higher body mass index, diabetes, higher education status, and smoking. The HRQoL scores were significantly higher in men. These findings are in accordance with previous research [5,13,24,25].

Symptoms including weakness, muscle pain, respiratory symptoms, voice disorders, taste/smell loss, and menstrual problems were more common in respondents with previous COVID-19 compared to controls. However, our study is a cross-sectional survey; therefore, the finding that symptoms persist beyond 12 weeks of the onset of COVID-19 infection cannot be attributed to post-COVID-19 symptoms or long COVID, especially since we did not have access to any information to exclude the alternative diagnosis. However, the findings are in line with published research, including studies with proven physiological data. For instance, Bostanci and colleagues evaluated respiratory function after COVID-19 infection in unvaccinated athletes and reported a decrease in respiratory function, even with mild COVID-19 disease [26]. A systematic review of observational studies identified symptoms in patients with COVID-19 or post-COVID-19, including general symptoms such as fatigue or asthenia including weakness, respiration such as cough and shortness of breath, pain such as muscle pain, and alteration of senses such as loss of taste or smell [27]. Li and coworkers found that COVID-19 could lead to transient changes such as prolonged cycles and decreased volume, which resolved to normal within one to two months postinfection [28]. Some studies linked COVID-19 vaccinations with menstrual changes making vaccination a confounder in our observations [29]. The impact of COVID-19 on the menstrual cycle is an area that requires further research.

The high prevalence of fatigue among respondents with COVID-19 signifies the need for rehabilitation programs tailored to the needs of patients; such programs were reported to be effective [30]. The management of fatigue and other post-COVID-19 symptoms is essential for reducing the negative impact of COVID-19 on the workforce [31]. 

The study evaluated post-COVID-19 symptoms through a self-reported questionnaire. 

### 4.1. Implications for Practice and Future Research

The impact of COVID-19 warrants allocating adequate healthcare resources for rehabilitation programs, management strategies for patients, and COVID-19 preventive measures. Although long COVID clinics were introduced in Saudi Arabia early in 2022, the extent of utilization, accessibility, and public awareness of these clinics is not fully examined in Arab countries. Furthermore, the effectiveness and cost-effectiveness of interventions delivered at these clinics need investigations.

### 4.2. Strength and Limitations

Our study is one of the few that compared HRQoL and fatigue between individuals previously infected with COVID-19 and controls. The study was not limited to hospitalized COVID-19 patients as much of the published evidence suggests. The study also contributed to a better understanding of the impact of sociodemographic characteristics, time to SARS-CoV-2 infection, and the number of SARS-CoV-2 infections on HRQoL. Nevertheless, the study has a few limitations. First, the cross-sectional nature of the data precludes establishing a cause-and-effect relationship. Second, we collected data using an online survey introducing selection bias [32]. Furthermore, we used social media platforms, e.g., WhatsApp and Twitter, to advertise our study and invite participants, creating limited opportunities for recruiting older adults and those without social media accounts [30]. Third, the data collected using self-report carries a risk of inaccuracies or recall biases and the control participants could have subclinical COVID-19 infection. Fourth, control patients may have experienced subclinical SARS-CoV-2 infection. Last, we used a generic instrument to measure HRQoL instead of a more sensitive disease-specific tool. Previous research on HRQoL in COVID-19 patients commonly used generic HRQoL tools such as SF-36 and EQ5D [5,15,24]. The reason could be the lack of available disease-specific measures, although one has been recently developed in English [33]. Additionally, this tool is suitable for comparison with noninfected controls. 

## 5. Conclusions

COVID-19 effects could persist beyond the acute infection phase. These effects include changes in quality of life, fatigue, and persistence of physical symptoms. Longer follow-up studies are required to identify how long the effects of COVID-19 persist and the special care required for those patients.

## Figures and Tables

**Table 1:** Comparison of baseline characteristics between patients with post-COVID-19 vs. control.

	No COVID-19 Disease (*n* = 565)	Post-COVID-19 Disease (*n* = 400)	*p*-Value
Female	379 (67.08%)	251 (62.75%)	0.164
Age category			0.001
18–25 year	159 (28.14%)	87 (21.75%)	
26–35 year	169 (29.91%)	88 (22%)	
36–45 year	135 (23.89%)	121 (30.25%)	
46–55 year	68 (12.04%)	74 (18.50%)	
56–65 year	29 (5.13%)	28 (7%)	
66–75 year	4 (0.71%)	2 (0.50%)	
More than 75 years	1 (0.18%)	0	
BMI (Kg/m^2^)	25 (21–29)	26 (23–29)	0.009
Smokers			0.505
Never	483 (85.49%)	331 (82.75%)	
Current smokers	71 (12.57%)	59 (14.75%)	
Ex-smoker (more than one month)	11 (1.95%)	10 (2.5%)	
Marital status			˂0.001
Single	268 (47.43%)	132 (33%)	
Married	260 (46.02%)	243 (60.75%)	
Divorced/widow/separated	37 (6.55%)	25 (6.25%)	
Country of residence			0.001
Saudi Arabia	476 (84.25%)	366 (91.50%)	
Outside Saudi Arabia	89 (15.75%)	34 (8.50%)	
Employment			˂0.001
Do not work	277 (49.03%)	125 (31.25%)	
Employee	132 (23.36%)	187 (46.75%)	
Business owner	33 (5.84%)	15 (3.75%)	
Student	93 (16.46%)	47 (11.75%)	
Retired	30 (5.31%)	26 (6.50%)	
Highest education level			˂0.001
High school or lower	224 (39.65%)	93 (23.25%)	
University graduate	284 (50.27%)	225 (56.25%)	
Postgraduate studies	57 (10.09%)	82 (20.50%)	
Current vaccination status			˂0.001
Unvaccinated	80 (14.16%)	21 (5.25%)	
One dose	17 (3.01%)	7 (1.75%)	
Two doses	153 (27.08%)	89 (22.25%)	
Three doses	301 (53.27%)	273 (68.25%)	
More than three doses	14 (2.48%)	10 (2.50%)	
Vaccine type			˂0.001
Moderna	5 (0.88%)	9 (2.25%)	
Pfizer-BioNTech	256 (45.31%)	230 (57.50%)	
AstraZeneca	38 (6.73%)	19 (4.75%)	
Johnson	2 (0.35%)	1 (0.25%)	
Sinopharm	7 (1.24%)	0	
Other	2 (0.35%)	1 (0.25%)	
I don’t know	8 (1.42%)	4 (1%)	
Combined	167 (29.56%)	115 (28.75%)	
Diabetes mellitus			0.216
Yes	31 (5.49%)	32 (8%)	
No	515 (91.15%)	351 (87.75%)	
I don’t know	19 (3.36%)	17 (4.25%)	
Cardiovascular disease			0.073
No	480 (84.96%)	325 (81.25%)	
Yes	58 (10.27%)	60 (15%)	
I don’t know	27 (4.78%)	15 (3.75%)	
Pulmonary disease			0.714
No	484 (85.66%)	335 (83.75%)	
Yes	64 (11.33%)	51 (12.75%)	
I don’t know	17 (3.01%)	14 (3.50%)	

**Table 2:** Comparison of the current symptoms between post-COVID-19 and non-COVID-19 participants.

	No COVID-19 Disease (*n* = 565)	Post-COVID-19 Disease (*n* = 400)	*p*-Value
Weakness	84 (14.87%)	114 (28.50%)	˂0.001
Joint stiffness	55 (9.73%)	47 (11.75%)	0.316
Muscle pain	151 (26.73%)	153 (38.25%)	˂0.001
Tingling and numbness	166 (29.38%)	119 (29.75%)	0.901
Respiratory symptoms	27 (4.78%)	36 (9%)	0.009
Voice disorders	29 (5.13%)	52 (13%)	˂0.001
Hypo/hypertension	84 (14.87%)	70 (17.5%)	0.271
Imbalance	83 (14.69%)	79 (19.75%)	0.038
Sexual dysfunction	26 (4.60%)	30 (7.50%)	0.058
Taste loss	24 (4.24%)	37 (9.25%)	0.002
Smell loss	28 (4.96%)	51 (12.75%)	˂0.001
Headache	197 (34.87%)	157 (39.25%)	0.164
Bowl symptoms	116 (20.53%)	91 (22.75%)	0.408
Urinary symptoms	63 (11.15%)	50 (12.50%)	0.521
Visual symptoms	104 (18.41%)	73 (18.25%)	0.950
Cardiac problems *	22 (3.89%)	21 (5.25%)	0.314
Menstrual problems (For women)	70/379 (18.47%)	78/251 (31.08%)	˂0.001
Pregnancy problems **	20/379 (5.28%)	14/251 (5.58%)	0.870
Dyspnea			0.116
Grade 0–I	420 (74.34%)	279 (69.75%)	
Grade II–IV	145 (25.66%)	121 (30.25%)	

* Cardiac problems included palpitation, chest pain or discomfort, dizziness, and syncope. ** Pregnancy problems included pain, bleeding, hypertension, diabetes, pre-eclampsia, and eclampsia.

**Table 3:** Comparison of the SF-36 domains between post-COVID-19 and non-COVID-19 participants (data were presented as median and interquartile limit).

	No COVID-19 Disease (*n* = 565)	Post-COVID-19 Disease (*n* = 400)	*p*-Value
Physical function	85 (50–95)	80 (50–95)	0.157
Role physical	100 (50–100)	75 (25–100)	0.045
Body pain	74 (54–100)	74 (51–100)	0.079
General Health	62 (55–75)	62 (52–75)	0.740
Vitality	55 (45–65)	50 (35–60)	˂0.001
Social functioning	75 (50–100)	75 (50–88)	0.129
Role emotional	33 (0–100)	67 (0–100)	0.010
Mental health	56 (44–72)	56 (44–70)	0.372
Reported health change	3 (2–3)	3 (2–4)	˂0.001
Physical-component summary	49.70 (41.61–55.62)	48.87 (40.52–55.08)	0.171
Mental-component summary	43.40 (35.53–50.32)	41.46 (32.55–49.36)	0.014

## Data Availability

The dataset that support the findings of this study are available upon reasonable request to the corresponding author.

## Appendix A

**Table A1:** Quality of life in patients infected with COVID-19 once vs. more than once (data were presented as median and interquartile limit).

	Once (*n* = 328)	More than Once (*n* = 72)	*p*-Value
Physical function	80 (50–95)	75 (58–95)	0.988
Role physical	88 (25–100)	50 (0–100)	0.049
Body pain	74 (52–100)	73 (41–92)	0.073
General Health	62 (55–75)	60 (45–72)	0.031
Vitality	50 (35–60)	50 (40–60)	0.788
Social functioning	75 (50–100)	63 (50–88)	0.090
Role emotional	67 (0–100)	33 (0–100)	0.007
Mental health	56 (44–72)	54 (42–68)	0.421
Reported health change	3 (2–4)	3 (2–4)	0.421
Physical-component summary	49 (41–55)	47 (39–55)	0.326
Mental-component summary	43 (33–50)	37 (31–45)	0.049

**Table A2:** Comparison of the SF-36 domains at different time periods after COVID infection (data were presented as median and interquartile limit).

	<6 Months (*n* = 85)	6–12 Months (*n* = 151)	12–18 Months (*n* = 60)	18–24 Months (*n* = 46)	>24 Months (*n* = 58)	*p*-Value
Physical function	85 (60–95)	75(55–95)	83 (50–95)	80 (50–90)	73 (35–95)	0.335
Role physical	100 (50–100)	75 (0–100)	88 (25–100)	88 (25–100)	88 (25–100)	0.479
Body pain	74 (51–100)	74 (52–100)	74 (62–100)	74 (41–100)	74 (52–100)	0.557
General Health	60 (52–72)	62 (52–77)	62 (57–76)	57 (52–72)	67 (57–77)	0.140
Vitality	50 (40–60)	50 (35–60)	50 (38–60)	50 (40–60)	50 (40–60)	0.662
Social functioning	75 (50–100)	63 (50–88)	75 (50–100)	63 (50–75)	75 (50–100)	0.170
Role emotional	67 (0–100)	67 (0–100)	100 (33–100)	67 (0–100)	67 (0–100)	0.719
Mental health	52 (44–68)	56 (40–72)	56 (40–66)	60 (48–72)	58 (44–72)	0.510
Reported health change	3 (2–4)	3 (2–4)	3 (2–4)	3 (2–4)	2.5 (1–3)	0.061
Physical-component summary	50 (42–56)	48 (39–55)	49 (43–55)	51 (40–55)	46 (40–52)	0.394
Mental-component summary	40 (32–49)	41 (32–49)	41 (34–49)	43 (34–48)	44 (33–51)	0.703

**Table A3:** Factors affecting SF-36 physical- and mental-component summary in all cohorts.

	β (95% CI)	*p*-Value
Physical-components summary		
Male	2.78 (1.58–3.97)	˂0.001
BMI	−0.16 (−0.26–−0.06)	0.003
Age Category	−0.62 (−1.11–−0.122)	0.015
Mental-components summary		
Age category	1.85 (1.25–2.44)	˂0.001
COVID-19 infection	−1.92 (−3.36–−0.48)	0.009
Male	3.40 (1.80–4.99)	˂0.001
Smoking	−2.27 (−3.67–−0.87)	0.001
Highest education degree	−1.39 (−2.49–−0.29)	0.013
Diabetes mellitus	−1.24 (−2.40–−0.08)	0.036

**Table A4:** Relationship between the current symptoms and physical- and mental-component summary among the post-COVID-19 infected participants.

	β (95% CI)	*p*-Value
Physical-component summary		
Muscle weakness	−4.07 (−6.05–−2.10)	˂0.001
Vision problems	−4.02 (−6.21–−1.83)	˂0.001
Urinary symptoms	−3.74 (−6.27–−1.20)	0.004
Joint stiffness	−4.43 (−7.14–−1.71)	0.001
Headache	−2.23 (−3.96–−0.50)	0.012
Loss of taste	3.28 (0.40–6.16)	0.026
Mental-component summary		
Weakness	−8.33 (−10.72–−5.95)	˂0.001
Headache	−2.56 (−4.78–0.35)	0.023

**Table A5:** The association between presenting symptoms and the physical- and mental-components summary of SF-36 score among the post-COVID-19 participants.

	β (95% CI)	*p*-Value
Physical-components summary		
Cough	0.62 (−1.26–2.49)	0.518
Shortness of breath	−2.46 (−4.44–−0.48)	0.015
Fever	−0.87 (0–2.76–1.03)	0.368
Loss of taste or smell	−0.32 (−2.13–1.48)	0.724
Mental-components summary		
Cough	0.50 (−2.85–1.85)	0.68
Shortness of breath	−4.07 (−6.56–−1.59)	0.001
Fever	−0.23 (−2.60–2.15)	0.852
Loss of taste or smell	−1.35 (−3.61–0.92)	0.243

**Table A6:** Factors affecting fatigue severity scale in all participants.

	β (95%CI)	*p*-Value
Cardiovascular disease	0.30 (0.05–0.54)	0.018
COVID-19 infection	0.44 (0.12–0.77)	0.008
Living outside Saudi Arabia	0.56 (0.07–1.04)	0.024
